# Sorafenib-based therapy in HER2-negative advanced breast cancer: Results from a retrospective pooled analysis of randomized controlled trials

**DOI:** 10.3892/etm.2014.1603

**Published:** 2014-03-05

**Authors:** QI-XING TAN, QING-HONG QIN, BIN LIAN, WEI-PING YANG, CHANG-YUAN WEI

**Affiliations:** Breast Surgery Department, Tumor Hospital, Guangxi Medical University, Nanning, Guangxi 530021, P.R. China

**Keywords:** advanced breast cancer, sorafenib, meta-analysis

## Abstract

A standard systemic therapy for patients with human epidermal growth factor receptor 2 (HER2)-negative advanced breast cancer (ABC) is yet to be identified. Sorafenib has been developed for the treatment of solid tumors, including breast cancer, as an oral multikinase inhibitor with antiangiogenic and antiproliferative activity. The aim of the present study was to assess the efficacy and safety of sorafenib in patients with HER2-negative ABC by performing a meta-analysis. A literature search was applied to databases, including PubMed, EMBASE, the Cochrane Library Databases, American Society of Clinical Oncology and the European Society for Medical Oncology, with the search terms ‘advanced breast cancer’ and ‘sorafenib’ and relevant studies were selected for analysis. The data extracted from the selected studies included progression-free survival (PFS), time to progression (TTP), overall survival (OS) and overall response rate (ORR). Major adverse events (AEs) were also analyzed. A total of four randomized controlled trials containing 844 cases were identified. Combined results revealed that when compared with chemotherapy (or with anti-hormone receptor therapy) alone, sorafenib-based therapy significantly increased the PFS [hazard ratio (HR), 0.78; 95% confidence interval (CI), 0.54–1.02] and TTP (HR, 0.74; 95% CI, 0.50–0.97), but not the OS (HR, 0.95; 95% CI, 0.75–1.15) and ORR (relative risk, 1.19; 95% CI, 1.01–1.39). In addition, the incidence of grade 3/4 AEs, including hand-foot skin syndrome, anemia, fatigue, rash and stomatitis, were significantly increased in patients that received sorafenib-based therapy. Therefore, the results from the current meta-analysis indicated that sorafenib-based therapy improved the PFS and TTP in patients with HER2-negative ABC, but not the OS and ORR. In addition, combination treatment was associated with increased toxicities and frequently required dose reductions.

## Introduction

Despite advances in diagnosis and treatment over several decades, breast cancer remains the highest cause of cancer mortality in females (1). Metastasis and recurrence often contribute to the poor clinical outcomes of breast cancer patients, and metastatic disease remains incurable. Combination chemotherapy regimens have demonstrated clinical benefits compared with single-agent regimens, but have also shown increased toxicity. Thus, the development of new drugs is critical to further improve and advance the treatment of breast cancer. For human epidermal growth factor receptor 2 (HER2)-positive advanced breast cancer (ABC), trastuzumab in combination with chemotherapy has become the standard first-line adjuvant treatment (2). However, with regard to HER2-negative ABC, there remain no effective targeted therapies. Therefore, the development and testing of novel agents that target pathways considered to be involved in the pathogenesis of HER2-negative ABC is required.

Sorafenib is an oral multikinase inhibitor that exhibits antiangiogenic and antiproliferative activity. The targets of sorafenib include the Ras/Raf/mitogen-activated protein kinase (MAPK) signaling pathway, platelet-derived growth factor receptor-α and β, vascular endothelial growth factor receptors (VEGFRs)-1, 2 and 3 and c-KIT and FLT3 kinases (3,4). These kinases play key roles in tumor cell proliferation, apoptosis and tumor angiogenesis (5). Compared with antiangiogenic monoclonal antibodies, including bevacizumab, a potential advantage of sorafenib is that it inhibits other receptors and intracellular signals involved in tumorigenesis, potentially offering multiple pathway inhibition. Preclinical studies have demonstrated that sorafenib decreases tumor cell proliferation *in vitro*, inhibits angiogenesis and induces apoptosis (6,7). In addition, sorafenib has been approved for the treatment of advanced renal cell (RCC) and unresectable hepatocellular carcinomas (HCC) (8,9). With regard to breast cancer, several randomized controlled clinical trials (RCTs) have compared sorafenib with a placebo for the treatment of ABC, with four studies reporting their results (10–13). In order to improve the evaluation of the potential role of sorafenib in combination with known effective palliative treatments for HER2-negative ABC, a meta-analysis was conducted to assess the efficacy and safety of sorafenib treatment in patients with HER2-negative ABC.

## Materials and methods

### Literature search and selection

An extensive search of PubMed (www.ncbi.nlm.nih.gov/pubmed), EMBASE (www.elsevier.com/online-tools/embase), the Cochrane Library Databases (www.thecochranelibrary.com), American Society of Clinical Oncology (www.asco.org) and the European Society for Medical Oncology (www.esmo.org) was conducted up to September 2013, using the following search terms: ‘Advanced breast cancer’ and ‘sorafenib’. In addition, associated keywords and their synonyms were included in the search strategy and reference lists were scanned for additional publications. RCTs that investigated the efficacy of sorafenib in HER2-negative locally recurrent/metastatic breast cancer were considered to be eligible. Trials had to meet the following inclusion criteria. Firstly, all the patients had a confirmed pathological diagnosis of HER2-negative advanced/metastatic breast cancer and were randomly assigned to treatment. Secondly, sorafenib or sorafenib-based therapy was administered to the research group and compared with placebo or placebo-based therapy that was administered to the control group. Finally, the studies provided information on survival rates, including progression-free survival (PFS), time to progression (TTP), overall survival (OS) and data regarding the overall response rate (ORR) and adverse events (AEs).

### Data extraction and quality assessment

Data extraction was performed independently by two authors according to the aforementioned inclusion criteria. The following information was collected: First author, year of publication, methodological quality, number of patients, patient characteristics, hazard ratios (HRs) and 95% confidence intervals (CIs) for PFS, TTP and OS, data regarding AEs, details of subgroup analysis and the number of patients acquired overall response (the sum of complete and partial tumour responses to drugs) that was assessed with Response Evaluation Criteria In Solid Tumors.

The quality of each retrieved study was independently assessed by the two authors, in accordance with the instrument reported by Jadad *et al* (14). Firstly, it was determined whether the trial reported an appropriate randomization method (score 0–2). Secondly, it was determined whether the trials reported an appropriate blinding method (score 0–2) and finally, whether the trials reported withdrawals and dropouts (score 0–1). The final score ranged between 0 and 5 for each study with higher scores indicating better methodology.

### Statistical analysis

Statistical analyses were performed using Stata 12.0 statistical software (StataCorp LP, College Station, TX, USA) for meta-analysis. Survival outcome data, including PFS, TTP and OS, were polled using the time-to-event HRs and 95% CIs as the operational measures, while relative risk (RR) for overall response to treatment and odds ratios (OR) for various types of toxicity were also calculated. Statistical heterogeneity among the trials was analyzed using the χ^2^ test and the I^2^ measure of inconsistency; P<0.1 or I^2^>50% indicated significant heterogeneity. Fixed-effects models were used in all analyses unless heterogeneity existed (P<0.1 or I^2^>50%). All statistical tests were two-sided and P<0.05 was considered to indicate a statistically significant difference.

## Results

### Literature search

**Figure 1:**
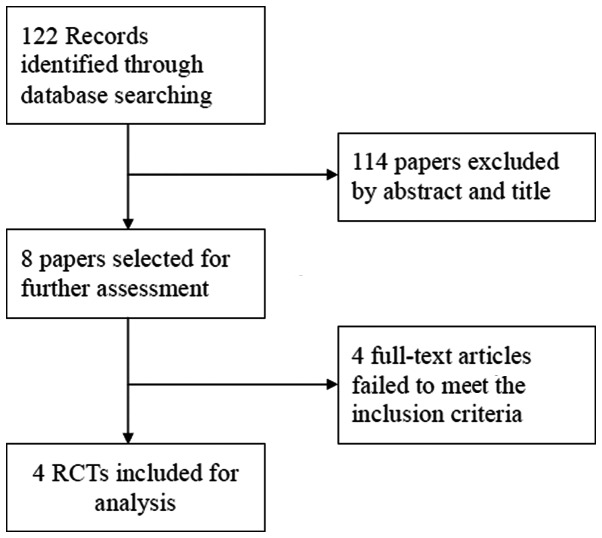
Flow chart demonstrating the identification and inclusion process of the trials selected for the meta-analysis. RCTs, randomized controlled trials.

A total of 122 potentially relevant studies were identified by the literature search. Following the review of each publication, 114 studies were excluded as they were not relevant to the aim of the present study, while eight relevant studies were selected for detailed evaluation. Four studies were excluded prior to analysis (15–18). Ultimately, four RCTs, including four phase IIB studies, were selected for analysis, involving a total of 844 patients (10–13). The study search process is shown in Fig. 1.

### Study characteristics

Characteristics of the four studies are shown in Table I. The four studies were phase IIB randomized, double-blind, placebo-controlled screening trials that were known collectively as Trials to Investigate the Effects of Sorafenib and were undertaken in patients with HER2-negative ABC. All the studies reported PFS and TTP data following treatment, as well as ORRs and AEs. Three studies reported the OS rates. The total number of included patients was 844. Among them, 426 patients received sorafenib-based therapy and 418 patients received placebo-based therapy. All the patients received treatment until disease progression, intolerable toxicity, consent was withdrawn or treatment was discontinued for other reasons.

### PFS

**Figure 2:**
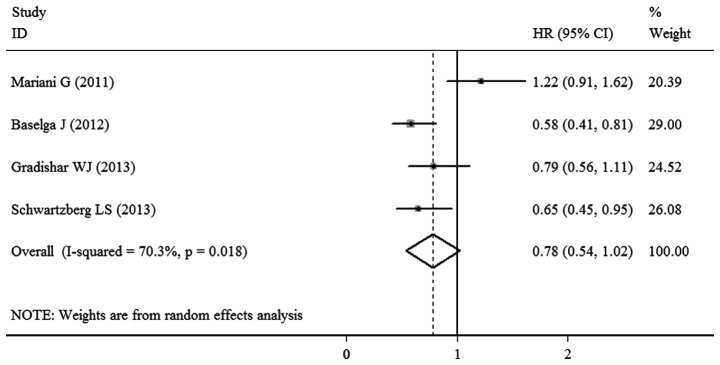
Forest plot for PFS comparing sorafenib- and placebo-based chemotherapies. The result indicates that sorafenib-based chemotherapy significantly increased the PFS time when compared with placebo-based chemotherapy in patients with HER2-negative ABC (HR, 0.78; 95% CI, 0.54–1.02). PFS, progression-free survival; ABC, advanced breast cancer; HR, hazard ratio; CI, confidence interval; HER2, human epidermal growth factor receptor 2.

Data regarding PFS were available in the four trials. Significant heterogeneity was observed among the studies (P=0.018; I^2^=70.3%), thus, the pooled HR for PFS was calculated using a random-effects model, with a result of 0.78 (95% CI, 0.54–1.02; P<0.00001; Fig. 2). This result demonstrated that there was a statistically significant difference between the sorafenib- and placebo-based therapy groups with regard to PFS.

### TTP

**Figure 3:**
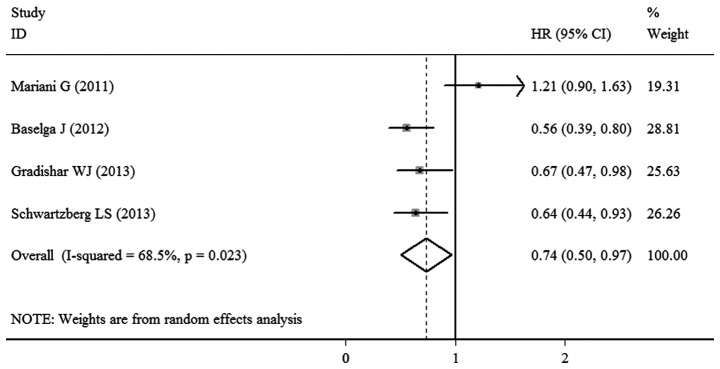
Forest plot for TTP comparing sorafenib- and placebo-based chemotherapies. The result indicates that sorafenib-based chemotherapy significantly increased the TTP when compared with placebo-based chemotherapy in patients with HER2-negative ABC (HR, 0.74; 95% CI, 0.50–0.97). ABC, advanced breast cancer; HR, hazard ratio; CI, confidence interval; HER2, human epidermal growth factor receptor 2; TTP, time to progression.

TTP data were available in the four trials. Due to the significant heterogeneity among the studies (P=0.023; I^2^=68.5%), a random-effects model was used. A statistically significant difference was observed between the sorafenib- and placebo-based therapy groups, as the pooled HR of the four RCTs was 0.74 (95% CI, 0.50–0.97; P<0.00001; Fig. 3).

### OS

**Figure 4:**
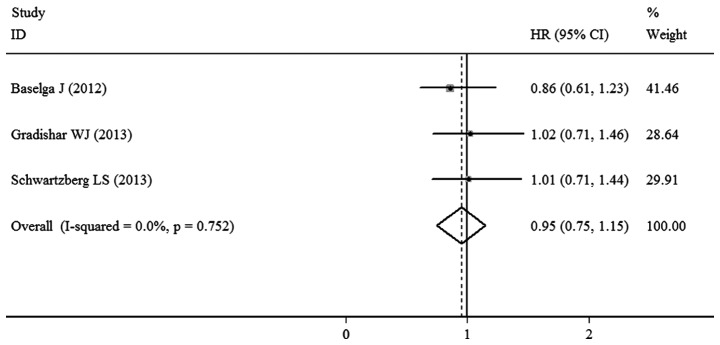
Forest plot for OS comparing sorafenib- and placebo-based chemotherapies. The result indicates that there was no significant difference between sorafenib- and placebo-based chemotherapies with regard to OS in patients with HER2-negative ABC (HR, 0.95; 95% CI, 0.75–1.15). OS, overall survival; ABC, advanced breast cancer; HR, hazard ratio; CI, confidence interval; HER2, human epidermal growth factor receptor 2.

OS was reported in three studies and the pooled HR of these RCTs was 0.95 (95% CI, 0.75–1.15; P<0.00001; Fig. 4). This result indicated that there was no significant difference between the sorafenib- and placebo-based therapy groups with regard to OS. No significant heterogeneity (P=0.752; I^2^=0.0%)was observed and the pooled HR for OS was calculated using the fixed-effects model.

### ORR

**Figure 5:**
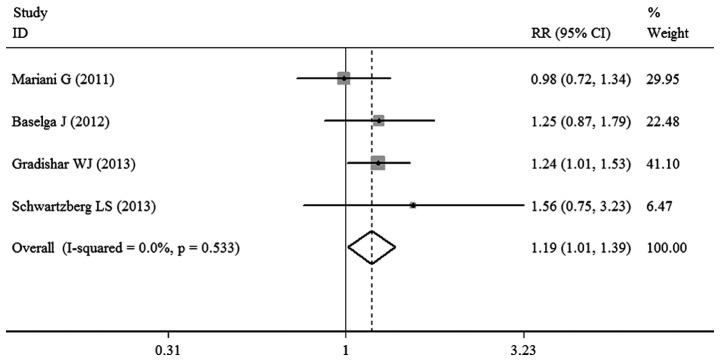
Forest plot for ORR comparing sorafenib- and placebo-based chemotherapies. The result indicates that there was no significant difference between sorafenib- and placebo-based chemotherapies with regard to ORR in patients with HER2-negative ABC (RR, 1.19, 95% CI, 1.01–1.39). ORR, overall response rate; ABC, advanced breast cancer; RR, relative risk; CI, confidence interval; HER2, human epidermal growth factor receptor 2.

ORR was reported in the four trials. There was no significant heterogeneity observed among the studies (P=0.533; I^2^=0.0%), thus, a fixed-effects model for meta-analysis was used. The pooled RR value was 1.19 (95% CI, 1.01–1.39; P=0.033; Fig. 5), demonstrating that sorafenib-based therapy did not significantly improve the ORR.

### AEs

All the trials provided information on multiple toxicities/AEs following treatment. However, only the incidence of major grade 3/4 AEs was assessed in the present study. The pooled result revealed that the risks of hand-foot skin syndrome, anemia, fatigue, rash and stomatitis were increased in the patients who received sorafenib-based therapy. However, there was no significant difference in other grade 3/4 AEs (Table II).

## Discussion

Sorafenib is an oral multikinase inhibitor that inhibits the Ras/Raf/MAPK signaling pathway, as well as platelet-derived growth factor receptors-α and β, VEGFRs-1, 2 and 3 and c-KIT and FLT3 kinases, to prevent tumor growth by antiangiogenic, antiproliferative and/or proapoptotic effects. The first phase I clinical trial evaluating oral sorafenib in patients with advanced solid tumors was initiated in July 2000 (19) and since then, sorafenib has been used to treat advanced RCC and HCC. With regard to breast cancer, two single-arm phase II studies demonstrated that sorafenib as a single agent, although well-tolerated, did not exhibit activity when measured by tumor shrinkage in patients with metastatic breast cancer (15–16). Thus, the role of sorafenib in the treatment of metastatic breast cancer has focused on combinations with standard therapies. For HER2-negative ABC patients, the lack of a specific target therapy has limited the benefits of treatment. Therefore, a new targeted agent is required that represents a novel approach to anticancer therapy, while also providing a higher therapeutic index. Several RCTs have investigated the efficacy of sorafenib in combination with systemic therapies for the treatment of HER2-negative ABC (by fluorescence *in situ* hybridization or immunohistochemistry), however, the results have been inconsistent. The SOLTI-0701 (11) and AC01B07 (13) trials demonstrated that anticancer activity was achieved when sorafenib was added to capecitabine and gemcitabine/capecitabine, respectively. By contrast, the NU07B1 (12) and FM-B07-01 (10) trials did not demonstrate a clinical benefit when sorafenib was added to paclitaxel and docetaxel/letrozole, respectively. Therefore, we hypothesized that it was necessary to integrate the data from these RCTs and evaluate the evidence with the aim of offering a more comprehensive insight into sorafenib-based therapy in patients with HER2-negative ABC. Thus, physicians and patients may make better-informed decisions regarding the most appropriate adjuvant therapy.

The results of the present meta-analysis demonstrated that sorafenib-based therapies provide a clinically modest, but statistically significant, PFS benefit (HR, 0.78; 95% CI, 0.54–1.02) in HER2-negative ABC patients. In addition, sorafenib therapy prolonged the TTP (HR, 0.74; 95% CI, 0.50–0.97), but failed to improved OS (HR, 0.95; 95% CI, 0.75–1.15) and ORR (RR, 1.19; 95% CI, 1.01–1.39). The majority of AEs associated with the addition of sorafenib were mild to moderate in severity. However, certain grade 3/4 AEs occurred more frequently in sorafenib-treated patients as compared with placebo-treated patients, including hand-foot skin syndrome, anemia, fatigue, rash and stomatitis.

Therefore, the development program for sorafenib in HER2-negative ABC has demonstrated encouraging activity when used in combination with selected chemotherapies, although the clinical benefit was relatively small.

In each of the RCTs, the primary endpoint was PFS and the starting sorafenib dose was 400 mg administered twice daily. However, the chemotherapies selected varied. The NU07B1 study (12) evaluated sorafenib in combination with paclitaxel as a first-line therapy. The study found that sorafenib-based chemotherapy improved disease control, but did not significantly improve the PFS, with a median PFS time of 6.9 months in sorafenib-treated patients and 5.6 months in placebo-treated patients (HR, 0.788; 95% CI, 0.558–1.112; P=0.1715). No significant difference was observed between the treatment groups with regard to OS, however, the addition of sorafenib was associated with statistically significant improvements in TTP and ORR. The SOLTI-0701 study (11) combined sorafenib with first- or second-line capecitabine. The addition of sorafenib to capecitabine resulted in a significant improvement in PFS when compared with placebo-based treatment (median PFS, 6.4 vs. 4.1 months; HR, 0.58; 95% CI, 0.41–0.81; P=0.001). Sorafenib was favored across the subgroups, including first- and second-line treatments, but there was no significant improvement for OS and ORR. In the AC01B07 trial (13), sorafenib was combined with gemcitabine or capecitabine in patients with HER2-negative ABC whose cancer had progressed during or following bevacizumab treatment. A clinically small, but statistically significant, PFS benefit was observed in the sorafenib patients (median PFS, 3.4 vs. 2.7 months; HR, 0.65; 95% CI, 0.45–0.95; P=0.02). Statistically significant improvements were was also observed in TTP, but there were no improvements to OS and ORR. These results support a potential role for sorafenib, when combined with gemcitabine or capecitabine, in the treatment of breast cancer that has progressed during or following the application of bevacizumab. The FM-B07-01 trial (10) investigated the potential benefit of sorafenib as an addition to the standard therapeutic approach in patients with HER-2 negative ABC, based on hormone receptor and visceral disease status. Patients with triple-negative breast cancer received docetaxel for a maximum of six cycles, patients with positive hormone receptor and visceral disease received docetaxel followed by letrozole and patients with positive hormone receptor and non-visceral disease received letrozole. However, there were no improvements to PFS, TTP or ORR associated with the addition of sorafenib to these regimens; OS data are pending.

Overall, the development program for sorafenib in HER2-negative ABC has demonstrated encouraging activity when used in combination with select chemotherapies. The current meta-analysis provides two types of clinically relevant evidence that further supports the development of sorafenib for the treatment of HER2-negative ABC. Firstly, the present study demonstrated activity for sorafenib when added to selected chemotherapeutic agents in various clinical scenarios, despite the PFS benefit being limited. In addition, as indicated by one of the RCTs, sorafenib may be able to overcome resistance in patients previously treated with bevacizumab by targeting multiple angiogenic pathways. Considering that the conditional approval of bevacizumab for breast cancer was revoked by the US Food and Drug Administration in 2011 (20), sorafenib may be developed as a novel approach to anticancer therapy in metastatic breast cancer. Secondly, although the incidence of specific AEs was increased in sorafenib patients, combination therapy was tolerable. Despite the high frequency of treatment discontinuations due to AEs, the sorafenib regimen exhibited a clinically manageable toxicity profile and no new or unexpected side effects were observed with this combination.

In view of the RCTs analyzed in the present study, there are specific questions that require further discussion and consideration. Firstly, the improvements in PFS and TTP with the addition of sorafenib did not translate into prolonged OS. This observation also occurred when bevacizumab was administered with standard chemotherapy for the treatment of metastatic breast cancer (21,22). One possibility is that the differences in postprogression treatments between the groups may have affected the OS outcome. In addition, it is possible that treatment with sorafenib adversely impacted the postprogression survival rate, by affecting tumor growth or toxicities. However, there was no evidence of postprogression mortalities associated with drug toxicity (23). Furthermore, dose interruptions and reductions were more common in the combination regimen, which may have affected the OS outcome. Other possible explanations include statistical chance or potential imbalances in baseline prognostic factors. Secondly, the variety of selected chemotherapeutic agents and the unselected patient population may have resulted in various outcomes. Subgroup analyses in each of the RCTs based on stratification factors and other baseline characteristics, including age, hormone receptor status and the presence of visceral disease, did not identify any patient subpopulations with statistically significant improvements. However, patients who had received prior adjuvant chemotherapy generally exhibited a greater PFS or TTP with the combination treatment. Validated biomarkers are likely to improve the understanding behind the variability in responses to antiangiogenic therapies across patient populations. Thus, the identification and validation of appropriate biomarkers for improved patient selection are required. The tumor growth-inhibitory effects of sorafenib may be attributed to the inhibition of tumor angiogenesis (24) and molecular markers involved in angiogenesis may be candidates. Preliminary biomarker evaluations have indicated that baseline soluble VEGFR and phosphorylated extracellular signal-regulated kinase levels are indicative of the sorafenib response in RCC (25) and HCC (26), respectively. However, at present, there are no proven biomarkers for selecting patients with ABC that are likely to benefit from antiangiogenic therapy (27). Thus, further study should be considered.

To the best of our knowledge, the present study is the first meta-analysis of RCTs comparing sorafenib- and placebo-based therapies for the treatment of HER2-negative ABC. As a meta-analysis, there are limitations that should be discussed. Firstly, although an individual patient data meta-analysis provides a more robust estimate of an association, obtaining individual patient data for the study was almost impossible. Therefore, a meta-analysis based on aggregated data from published literature was conducted. Secondly, the literature search was performed in limited databases. The total number of included studies and sample size were relatively small, which may have affected the validity of the meta-analysis to a certain extent. Possible publication bias is also a potential limitation of the present study, although this was not detected statistically. However, the four trials included were double-blind, randomized, placebo-controlled and performed on an intention-to-treat analysis. Therefore, the data extraction from these trials is reliable. In addition, a random-effects model was used to analyze PFS and TTP due to the significant heterogeneity observed among the studies. Since the random-effects model reduced the effect of large samples with better quality, this model was not as stable as the fixed-effects model.

In conclusion, the meta-analysis of the present study demonstrated that the addition of sorafenib to selected first or second-line chemotherapies provides statistically significant improvements in PFS and TTP for the treatment of HER2-negative ABC. However, the OS or ORR failed to improve significantly. The incidence of grade 3/4 AEs was generally higher in patients administered a combination of sorafenib and chemotherapy. Therefore, the sorafenib-based therapy regimen evaluated in the present study is not currently recommended for routine clinical practice in the treatment of HER-2 negative ABC, until further investigation and larger prospective clinical trials provide more data. Future issues for the development of sorafenib also include the identification and validation of appropriate biomarkers for improved patient selection.

## Figures and Tables

**Table I:** Baseline characteristics of the four eligible RCTs used in the meta-analysis.

Parameter	FM-B07-01	SOLTI-0701	NU07B1	AC01B07
Author	Mariani *et al* (10)	Baselga *et al* (11)	Gradishar *et al* (12)	Schwartzberg *et al* (13)
Publish year	2011	2012	2013	2013
Population	Italy, Germany, Poland, Russia	Spain, France, Brazil	India, USA, Brazil	USA
Phase	IIB	IIB	IIB	IIB
Sample size, n (S/P)	208 (111/107)	229 (115/114)	237 (119/118)	160 (81/79)
Age, years, S/P	NC	55.1/54.4	50.6/53.1	53.5/54.2
Therapy line	First	First/second	First	First/second
Treatment	SOR + DOC and/or LET vs. PLA + DOC and/or LET	SOR + CAP vs. PLA + CAP	SOR + PAC vs. PLA + PAC	SOR + GEM/CAP vs. PLA + GEM/CAP
Design	Parallel randomized	Parallel randomized	Parallel randomized	Parallel randomized
Blinding	Double-blind	Double-blind	Double-blind	Double-blind
Multicenter	Yes	Yes	Yes	Yes
Allocation concealment	Yes	Yes	Yes	Yes
ITT analysis	Yes	Yes	Yes	Yes
Survival analysis	PFS/TTP	PFS/TTP/OS	PFS/TTP/OS	PFS/TTP/OS
HR	Reported in text	Reported in text	Reported in text	Reported in text
Quality score	5	5	5	5

SOR, sorafenib; PLA, placebo; DOC, docetaxel; PAC, paclitaxel; GEM, gemcitabine; CAP, capecitabine; LET, letrozole; S, sorafenib group; P, placebo group; NC, not clear; ITT, intend-to-treat; RCTs, randomized controlled trials; HR, hazard ratio; PFS, progression-free survival; TTP, time to progression; OS, overall survival.

**Table II:** Meta-analysis results for the major grade 3/4 AEs.

		SOR group		PLA group			
					
AE	Evaluable trials, n	N1	A1, n (%)	N2	A2, n (%)	OR (95% CI)	P-value
Hypertension	4	417	7 (1.7)	414	7 (1.7)	0.99 (0.34–2.84)	0.098
Diarrhea	4	417	17 (4.1)	414	9 (2.2)	1.92 (0.85–4.37)	0.119
Hand-foot syndrome	4	417	127 (30.1)	414	24 (5.8)	7.83 (4.87–12.59)	0.000
Rash	4	417	24 (5.8)	414	2 (0.5)	7.73 (2.50–23.95)	0.000
Stomatitis	3	306	15 (4.9)	307	0 (0.0)	11.60 (2.18–61.64)	0.004
Asthenia	3	338	14 (4.1)	337	7 (2.1)	1.98 (0.81–4.84)	0.135
Fatigue	3	306	24 (7.8)	307	12 (3.9)	2.18 (1.04–4.57)	0.039
Mucositis	3	306	6 (2.0)	307	5 (1.6)	1.19 (0.38–3.74)	0.763
Thrombocytopenia	3	306	13 (4.2)	307	6 (2.0)	1.81 (0.19–17.19)	0.607
Neutropenia	4	417	54 (13.0)	414	44 (10.6)	1.26 (0.81–1.96)	0.302

N1, total number of patients evaluable for assessment of grade 3/4 AEs in the SOR group; A1, total number of patients who developed toxicities in the SOR group; N2, total number of patients evaluable for assessment of main AEs in the PLA group; A2, total number of patients who developed toxicities in the PLA group; SOR, sorafenib; PLA, placebo; OR, odds ratios; CI, confidence interval; AE, adverse event.
